# Supplementary material for: Implicit neural image field for biological microscopy image compression
Source: Nat Comput Sci. 2025 Oct 10;5(11):1041–50. doi: 10.1038/s43588-025-00889-4 (PMC12638249; doi:10.1038/s43588-025-00889-4)
Supplement: Supplementary file 1 — Supplementary text, notes, Tables 1–9 and Figs. 1–14. [file 43588_2025_889_MOESM1_ESM.pdf]

nature computational science

Supplementary information

https://doi.org/10.1038/s43588-025-00889-4

Implicit neural image field for biological microscopy image compression

In the format provided by the authors and unedited

1 Supplementary Tables

Supplementary Table 1: Overview of Datasets and Experiments in the Main Text. This section presents a concise summary of the case studies included in the main text. The study encompasses a diverse range of data derived from various imaging modalities and biological structures, aiming to substantiate our conclusions across a wide array of applications.

Figure	Dataset	Task	Biological Structure	Microscope
2	WTC-11 hiPSC Single-Cell Image	Compression	25 cellular structures	Spinning-disk confocal microscope
3	1-TNBC	Compression	breast tumour	Multi-channel fluorescence microscopy
4	Mouse Organoids	Compression	Organoids	Timelapse dual oblique plane microscopy
5	Troponin I	Compression (segmentation guidance)	Type 1 TNNI1	Spinning-disk confocal microscope
6	Tribolium	Compression (denoising guidance)	Whole embryo of Tribolium castaneum	Confocal

Supplementary Table 2: FOVs from the *hiPS single cell dataset V1* Used in Our Experiment. We listed the FOV ID and the visualized slice number (Z-depth) of the samples used in our 3D volumetric data compression task.

FOV ID	Demonstrated Z value
10728	27
2299	35
142674	28
6030	34
10684	26
13309	28

Supplementary Table 3: **The Visualization Slice Number of the 5D Mouse Organoid Dataset.** We listed the visualized slice number (C-channel, T-time, and Z-depth) of the samples used in our 5D data compression task.

Demonstrated C value	Demonstrated T value	Demonstrated Z value
1	32	60
1	32	80
1	32	120
2	32	60
2	32	80
2	32	120
1	97	60
1	97	80
1	97	120
2	97	60
2	97	80
2	97	120

Supplementary Table 4: **Data Usage in Noisy Data Compression Task.** We listed the file name and noise level of the samples used in our nosiy data compression task.

File Name	Laser Power	Dimensions
`nGFP_0.1_0.2_0.5_20_13_late`	Low	665 x 773 x 48
`nGFP_0.1_0.2_0.5_20_14_late`	Low	486 x 954 x 45
`nGFP_0.1_0.2_0.5_20_13_late`	Very Low	665 x 773 x 48
`nGFP_0.1_0.2_0.5_20_14_late`	Very Low	486 x 954 x 45

Supplementary Table 5: **Overview of Baseline Methods and Code Availability** In our released code, we provided the implementation of our baseline methods. We listed the source code we referenced.

Method	GPU	Language/Source
HEVC	NO	C/C++ / https://www.videolan.org/developers/x265.html
SIREN	YES	Python / https://github.com/lucidrains/siren-pytorch
INIF (ours)	YES	Python / https://github.com/PKU-HMI/INIF

2 Additional Information

2.1 Background

2.1.1 Implicit Neural Compression

Implicit Neural Representations (INRs), introduced by [1–4], have emerged as a powerful method for data representing, such as images [5], videos [6, 7], and audios [8, 9]. INRs leverage neural networks to approximate a compact, continuous function, enabling efficient representation of high-dimensional data. This ability has led to the

Supplementary Table 6: **Quantitative Results Comparison for all Main Text Experiments.** The best metrics are colour coded into red, and the second-best metrics are colour coded into blue.

Exp. #	Experiment	Target ratio	Metric	HEVC	SIREN	INIF
Fig. 2	Volumetric data test<br>3D, 16 bit, 25 samples	128	PSNR<br>SSIM	76.25<br>0.9926	76.8<br>0.9965	78.3<br>0.998
		256	PSNR<br>SSIM	75.75<br>0.992	76.5<br>0.9961	78.2<br>0.9976
Fig. 3	Multiplex data test<br>3D, 16 bit, 6 channels	256	PSNR<br>SSIM	47.32<br>0.9832	45.62<br>0.9811	48.04<br>0.9853
Fig. 4	5D data test<br>5D, 8 bit	256	PSNR<br>SSIM	71.02<br>0.9432	79.89<br>0.9822	81.21<br>0.9887
Fig. 5	App. approp. guidance<br>Segmentation task	256	PSNR<br>SSIM<br>Dice	73.15<br>0.9968<br>0.702	73.7<br>0.9921<br>0.689	74.79<br>0.9952<br>0.818
Fig. 6	App. approp. guidance<br>Low laser power	256	PSNR<br>SSIM<br>LPIPS	24.43<br>0.2445<br>0.9871	24.46<br>0.242<br>0.9726	18.06<br>0.051<br>0.9167
	App. approp. guidance<br>Very low laser power		PSNR<br>SSIM<br>LPIPS	75.77<br>0.9999<br>0.7756	79.37<br>0.9999<br>0.7625	75.19<br>0.9998<br>0.7011
Ext. Fig. 1	CODEC prior guidance<br>Speed up compression	256	PSNR<br>SSIM<br>Energy (kWh)	76.26<br>0.9999<br>0.0001	74.18<br>0.9998<br>0.15	77.75<br>0.9999<br>0.02

development of implicit neural compression, which has shown promising results for efficient image compression by encoding images as compact neural network weights [10]. The INR network training pipeline of INIF is shown in **Supplementary Figure 1**.

The core idea behind implicit neural compression is to store all information implicitly in the network weights θ. During training, it trains a neural network f_θ parameterized by weights θ, typically a coordinate-based multi-layer perceptron (MLP) [11], to map low-dimensional coordinate inputs $\mathbf{x} \in \mathbb{R}^n$ (e.g., spatial coordinates for images or space-time coordinates for videos) to the corresponding high-dimensional signal values $\mathbf{y} \in \mathbb{R}^m$ (e.g., grayscale pixel values for bioimages or RGB-alpha values for videos with transparency):

$$f_\theta : \mathbb{R}^n \to \mathbb{R}^m, \quad \mathbf{y} = f_\theta(\mathbf{x}) \tag{1}$$

During training, the neural network learns to overfit the underlying data distribution by minimizing a reconstruction loss $\mathcal{L}$ between the network output and the target data:

$$\min_\theta \mathcal{L}(f_\theta(\mathbf{x}), \mathbf{y}^{\text{target}}) \tag{2}$$

Normally, the reconstruction loss $\mathcal{L}$ is chosen to use Mean Squared Error(MSE) to measure the similarity of the ground-truth target and the INRs output. Once the network is trained, we only need to store the network weights θ because they constitute a continuous and compressible representation of the data. To reconstruct the original

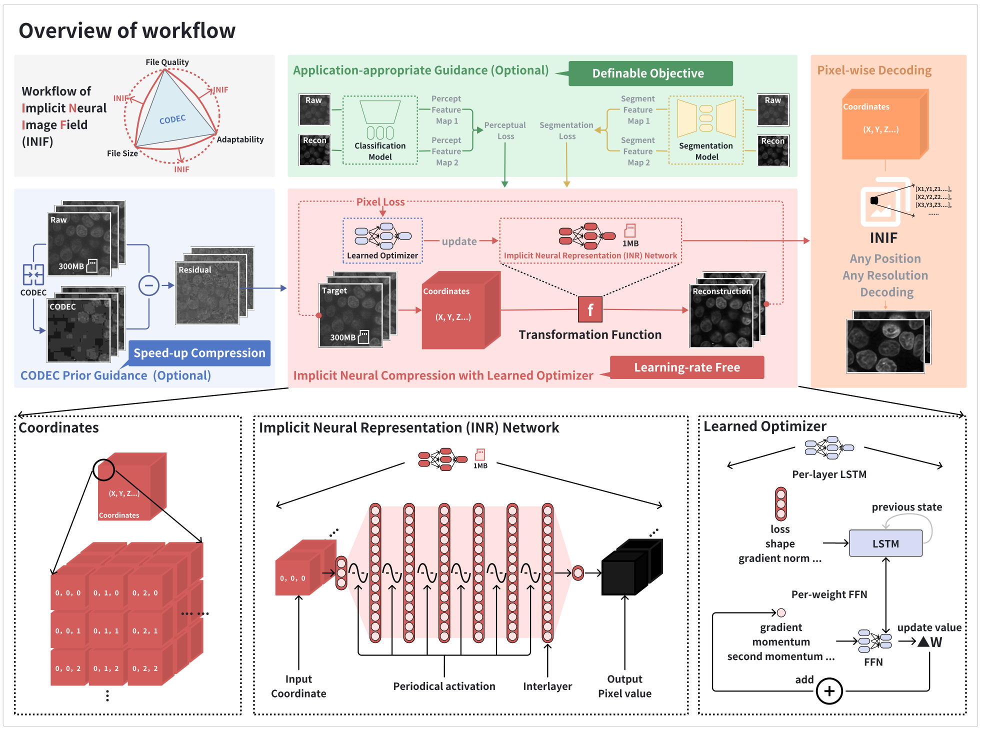

Supplementary Figure 1: **Pipeline for INR-based Compression .** Starts with creating the coordinates with the same shape as the data, followed by iterative passing those coordinates into a small INR network. The output of the INR network would be the predicted pixel values, which are further compared with the raw data value for back-propagation calculation. Values are then sent to the learned optimizer to predict the updated value for each parameter. More details of the learned optimizer can be found in **Supplementary Section** 2.2

data or decompress it one simply evaluates the trained function f_θ at the desired input coordinates $\mathbf{x}$, thereby obtaining the corresponding signal values $\mathbf{y}$.

However, implicit neural compression also faces challenges. The optimization process to fit the network to the target data can be computationally expensive, especially for high-resolution images or videos. Additionally, the compression performance may vary depending on the data content and the architecture of the neural network used. To address these challenges, recent works have explored various techniques to improve the efficiency and performance of implicit neural compression. These techniques include improving network structures [12–14], selecting appropriate activation functions [3, 15, 16], and employing embeddings [17, 18].

2.2 Learned Optimizer

2.2.1 Learned Optimization

In traditional machine learning optimization methods, hand-designed optimizers are typically used to train networks. A significant issue with hand-designed optimizers is the need for meticulous tuning of hyperparameters across different tasks. Without such tuning, training can fail or become trapped in local optima. The learned optimizer is a novel approach that attempts to use neural networks to predict the updates for the network being trained. The concept of meta-learning update rule can be traced back to the work of Bengio et al. [19]and Runarsson and Jonsson [20], which both trained simple neural networks to learn simple update rule.

In 2016, Andrychowicz et al. [21] meta-trained an RNN-parameterized learned optimizer on deep learning tasks by backpropagating through the optimization process, and this work sparked a significant wave of research, leading to the development of various new techniques. Wichrowska et al. [22] introduced hierarchical learned optimizers. Their approach involved meta-training these optimizers on a broad range of synthetic tasks. More recently, Metz et al. [23] extended this line of research by focusing on a more realistic task distribution and enhancing the learned optimizer architecture [24]. Finally, in our work, we used the Versatile Learned Optimizers (VeLO) provided by Metz et al. [25], which was meta-trained at a far greater scale than the previous investigation.

2.2.2 VeLO

We can define the optimization problem as follows:

$$\phi_{t+1} = \phi_t - U(\phi_t, \nabla_{\phi_t}; \theta) \tag{3}$$

where ϕ represents the parameters of the INR being optimized, ∇ denotes the gradient of the loss function of the INR network obtained through backpropagation, θ are the parameters of VeLO, and U is the update predicted by VeLO.

As shown in **Supplementary Figure 1**, the core module of VeLO is hierarchical and consists of two components: a per-tensor LSTM and a per-parameter MLP.

The per-tensor LSTM is composed of 512 hidden units and is responsible for managing the overall training features. It serves as a hypernetwork for the per-parameter MLP by providing individualized parameters for the per-parameter MLP. Its input features reflect the overall training state and the statistical characteristics of each parameter, including the following features: (1) Fraction of training remaining: This feature uses the current iteration t and he total target iterations T to compute a set of training soft progress values using the formula:

$$\tanh(10 \times (t/T - s)) \tag{4}$$

(2) Loss features: These features use the exponential moving average and running minimum of the loss to construct features that reflect the training trend, independent of the loss magnitude. The computed values range from $[-1, 1]$, where negative values

roughly correspond to decreasing loss, positive values indicate increasing loss, and zero indicates no change in loss, (3) the first and second moment of momentum features, and (4) the rank of the tensor. Finally, the per-tensor LSTM outputs value d and scalar learning rate c_{lr}, which are used to update the per-parameter MLP as follows:

$$\Delta\theta_p = 0.001 \times d \times \exp(0.001 \times c_{lr})\|\theta_p\|_2 \tag{5}$$

The per-parameter MLP operates specifically on feature sets, using a minimal MLP (2-hidden layers, 4-hidden units), which maintains computational efficiency with high performance. For all parameters in the INR we want to optimize, VeLO initializes a unique hyper MLP (hMLP). The initialization values are determined by a uniform distribution and meta learned parameter weights. The hMLP predicts an update U_θ after receiving the gradient, momentum, second moment, and other features of the INR network, to update the INR network weight ϕ in **Supplementary Equation 3**.

2.3 CODEC

2.3.1 Hybrid coding framework

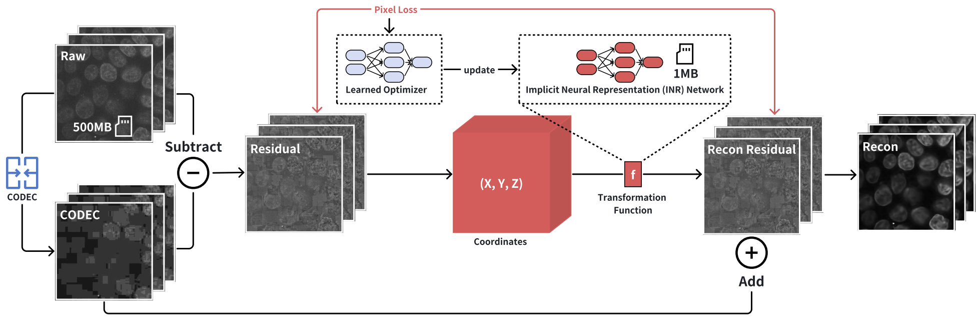

Supplementary Figure 2: **CODEC prior for speed-up INR compression.** CODEC can produce a reasonable result at a relatively cheap cost. Consider this as a starting point, INR networks are utilized as the differentiable adapter to further adapt the result.

One of our designs uses CODEC priors as a solid starting point before INR compression (**Supplementary Figure 2**). Current commercial coding standards for image and video compression, such as H.264/AVC [26] and H.265/HEVC [27], are based on hybridizing a broad range of algorithms for predictive coding. Those algorithms assume the presence of spatial and temporal redundancy in pictures and videos. During the compression process, the CODEC uses pixels in adjacent areas to predict the current area and then records the residual between the true value and the predicted value to achieve data compression. As shown in **Supplementary Figure 3**, the HEVC standard follows the hybrid coding framework. The encoding steps of HEVC are

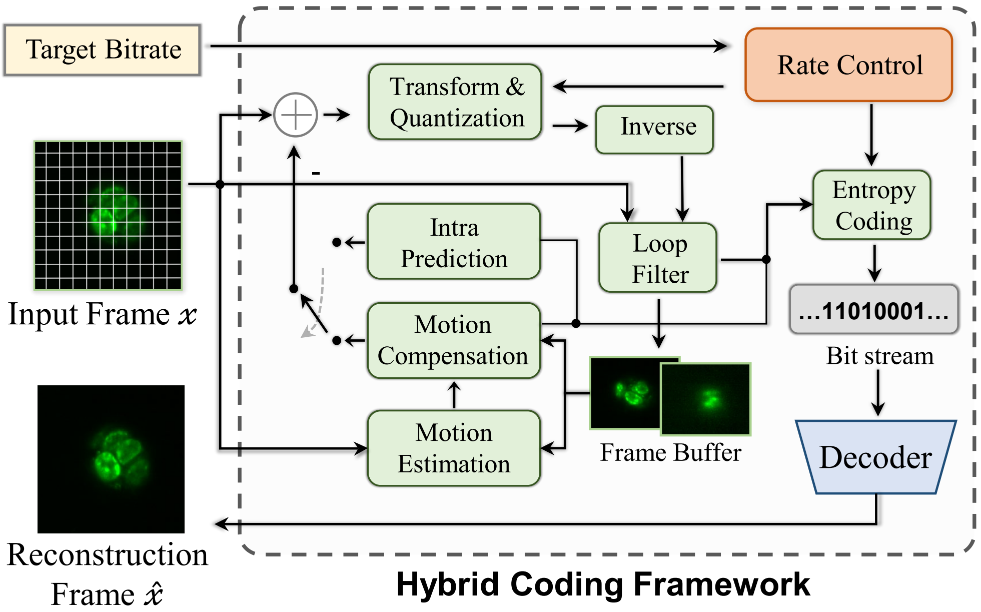

Supplementary Figure 3: **HEVC Hybrid Coding Framework.** Inputting multi-frame data, the HEVC encoder performs intra-frame and inter-frame prediction, respectively, to eliminate redundant information within and between images.

mainly divided into block division, intra-prediction, inter-prediction, transformation, quantization, post-processing filtering, and entropy coding.

During the execution of encoding, the HEVC CODEC considers the results of different encoding tools and encoding parameters:

$$\arg\min_{\theta}(D + \lambda \cdot R), \tag{6}$$

where D represents the coding distortion, R represents the bitrate required to encode the information, and λ represents the Lagrange multiplier to balance D and R. θ represents the combination of coding tools and coding parameters. CODEC aims to minimize **Supplementary Equation 6**. This process is called Rate-distortion Optimization (RDO) [28, 29] and is the basic principle of CODEC encoding.

For each frame of input data, the HEVC CODEC first divides it into Coding Tree Units (CTUs) according to the predefined fixed size. Then, the CODEC further divides the units according to the complexity of the image texture to obtain Coding Units (CUs), Prediction Units (PUs), and Transform Units (TUs) for subsequent operations. Considering the existence of spatial redundancy, which means the pixel values of adjacent areas are similar, the HEVC CODEC can predict the pixel value of the current PU with reference to the encoded intra-pixels. When pictures are taken continuously, there are also similarities between pixels at adjacent positions in adjacent frames. Therefore, the HEVC CODEC can also use units of adjacent frames to

perform inter-frame prediction on the current PU. The residual between the predicted value and the true value of the unit will be transformed to the frequency domain by CODEC, and quantization with different step sizes will be performed according to different frequency bands. This reduces the amount of data that needs to be stored while minimizing perceptible distortion. Quantization introduces distortion to the image. Therefore, the CODEC performs in-loop filtering on the reconstructed image to alleviate artificial artifacts such as blocking and ringing introduced during the lossy compression process. Finally, all the above coding modes and data information are written into the bit stream in a lossless manner by the entropy coding module.

2.3.2 Rate Control

As an important tool for CODEC control, the rate control algorithm can make the bitrate of the encoder output stream consistent with the specified target, thereby realizing the function of controlling the compression rate [30]. Before encoding, the target bitrate for rate control can be obtained based on the original size of the data and the target compression rate. Given a target bitrate, the rate control algorithm maps it to encoding parameters for different frames and CTUs, such as Quantization Parameter (QP) and λ in **Supplementary Equation 6**. QP achieves different distortions by controlling the quantization step size of the unit. λ achieves different compression rates by adjusting the weight of the unit's distortion and bitrate during the RDO process.

Basically, rate control is divided into bit allocation and coding parameter derivation. Based on the target bitrate, frame size, and frame rate, the average number of bits consumed per frame can be calculated. Taking further into account the importance of different frames in the reference process and the actual consumption and surplus of bits by encoded frames, bits are adaptively allocated to frames and CTUs. For the coding unit, the bitrate and distortion have an inverse relationship as shown in **Supplementary Figure 4**. In HEVC, the relationship between R and D is modeled as a hyperbolic function:

$$D(R) = C \cdot R^{-K}, \tag{7}$$

where C and K are model parameters, which vary with coding unit content characteristics. Combining **Supplementary Equation 6** and **Supplementary Equation 7**:

$$\lambda = \frac{\partial D}{\partial R} = C \cdot K \cdot R^{-K-1} \triangleq \alpha \cdot R^{\beta}, \tag{8}$$

where α and β are model parameters that are dynamically updated to reflect the characteristics of the unit and the outcomes of the encoding process throughout the encoding period. With the help of **Supplementary Equation 8**, the bits are mapped to the encoding parameter λ to adjust the RDO. QP can also be further derived:

$$QP = c_1 \cdot \ln(\lambda) + c_2, \tag{9}$$

where c_1 and c_2 are respectively set to 4.2005 and 13.7122 in HEVC.

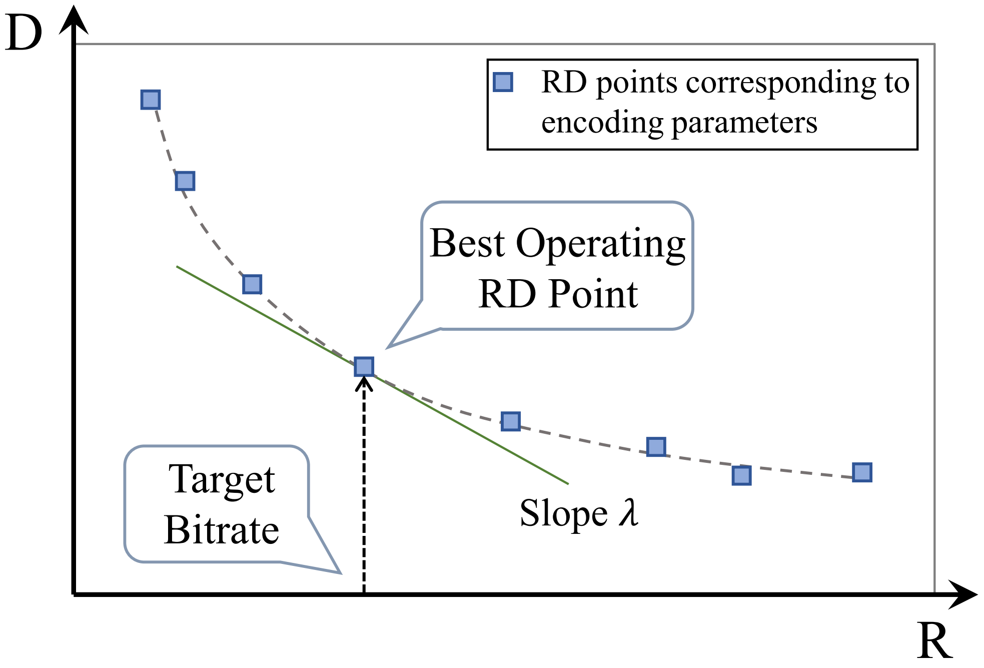

Supplementary Figure 4: **Rate-distortion Curve of Coding Unit.** The X-axis represents the bit-rate for encoding. Y-axis represents the distortion of the decoded signal. From the graph, we can see a negative relationship between bit-rate and distortion.

2.4 Main Text Experiments

2.4.1 Overview

For each of the described experiments, our method consists of the following steps:

1. **Image Normalization**: Before training, each image is normalized to ensure a consistent scale across different images. This step is critical as it helps to standardize the input data, improving the stability and performance of the training process.
2. **Training for Compression**: For every selected image, an INR model is individually trained to compress that image. This process involves optimizing the INR to overfit the image directly into the model's parameters.
3. **Reconstruction**: The trained INR models are then used to reconstruct the original images by feeding their coordinates. In particular, each INIF model is trained on a specific image, capturing its unique content (e.g., nuclei, microtubules). We also used the auto-adjustment function in ImageJ for all of our results (including baselines) to better demonstrate the visualization qualities.

2.4.2 Image normalization

For both the training and prediction phases, normalizing input images to a standard range is crucial. This is because the compressed image $\mathbf{y}$ predicted by our network and the corresponding ground-truth image $\mathbf{y}^{\text{truth}}$ typically differ significantly in the dynamic range of their pixel values. Normalization is commonly achieved by scaling the inputs to a specified minimum and maximum value. In our work, we employ min-max normalization [31] to scale each pixel value and coordinate within the specified range. The normalized image $N(\mathbf{y})$ and coordinate $N(\mathbf{x})$ are formulated as follows:

$$N(\mathbf{y}; \mathbf{y}_{\text{min}}, \mathbf{y}_{\text{max}}) = \frac{\mathbf{y} - \mathbf{y}_{\text{min}}}{\mathbf{y}_{\text{max}} - \mathbf{y}_{\text{min}}} \tag{10}$$

$$N(\mathbf{x}; \mathbf{x}_{\text{min}}, \mathbf{x}_{\text{max}}) = \frac{\mathbf{x} - \mathbf{x}_{\text{min}}}{\mathbf{x}_{\text{max}} - \mathbf{x}_{\text{min}}} \tag{11}$$

where pixel values are scaled to the range from 0 to 100, and coordinate values are scaled to the range from -1 to 1.

2.4.3 Image quality assessment

Assessing the quality of image compression is critical to ensure that the compressed images maintain a high degree of fidelity to the original. To this end, we utilize several metrics to assess the quality of our image compression:

1. **Pixel level loss**: We utilize Mean Squared Error (MSE) to quantify the average of the squares of the differences between the ground-truth image, $\mathbf{y}^{\text{truth}}$, and the compressed image, $\hat{\mathbf{y}}$. This metric is widely used in image processing to measure the quality of reconstructed images as it directly corresponds to pixel-wise intensity differences:
$$\text{MSE}(\mathbf{y}^{\text{truth}}, \hat{\mathbf{y}}) = \frac{1}{n} \sum_{i=1}^{n} (\mathbf{y}_i^{\text{truth}} - \hat{\mathbf{y}}_i)^2, \tag{12}$$
Here, n represents the total number of pixels in the image.
2. **The perceptual loss**: Perceptual loss [32] measures the difference in perceptual features between two images, capturing aspects that traditional pixel-based metrics might miss. Initially, we used the AlexNet [33] model, pre-trained on the Allencell [34] dataset, to extract high-level semantic features from both the original and compressed images. This approach helps in assessing how well the compression model preserves content that is perceptually significant to human observers:
$$\mathcal{L}_p = \sum_{i=1}^{L} \frac{1}{H_i W_i} \sum_{h=1}^{H_i} \sum_{w=1}^{W_i} \| w_i \cdot (\Phi_i(\hat{\mathbf{y}}) - \Phi_i(\mathbf{y}^{\text{truth}})) \|_2^2, \tag{13}$$
where Φ_i denotes the feature map obtained from the i-th layer of AlexNet, and w_i represents the weight factors for the respective layers. We use perceptual loss in our unsupervised, robust noisy data compression experiment. Listed in **Main Section 2.2**. The pipeline is shown in **Supplementary Figure 5**

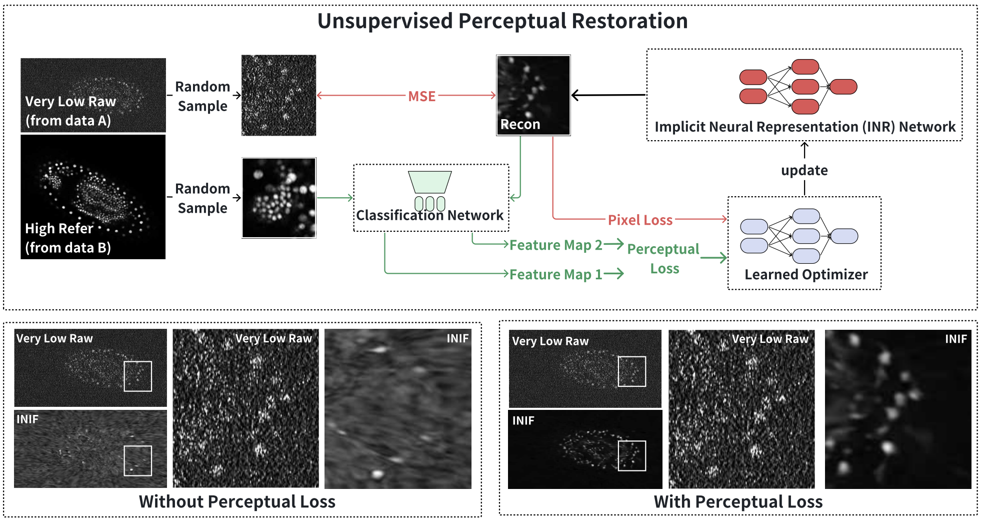

Supplementary Figure 5: **Unsupervised perceptual restoration.** The overall workflow is illustrated on the top section, while example compression results with and without the perceptual loss are shown in the bottom section.

3. **The structural similarity index measure (SSIM)**: SSIM [35] is an advanced metric that evaluates image quality by comparing the luminance $l(\mathbf{y}^{\text{truth}}, \hat{\mathbf{y}})$, contrast $c(\mathbf{y}^{\text{truth}}, \hat{\mathbf{y}})$, and structural $s(\mathbf{y}^{\text{truth}}, \hat{\mathbf{y}})$ similarities between the original $\mathbf{y}^{\text{truth}}$ and compressed images $\hat{\mathbf{y}}$. Unlike MSE, SSIM considers perceptual phenomena, making it more aligned with human visual perception:

$$\text{SSIM}(\mathbf{y}^{\text{truth}}, \hat{\mathbf{y}}) = l(\mathbf{y}^{\text{truth}}, \hat{\mathbf{y}}) \cdot c(\mathbf{y}^{\text{truth}}, \hat{\mathbf{y}}) \cdot s(\mathbf{y}^{\text{truth}}, \hat{\mathbf{y}}), \tag{14}$$

where:

$$l(\mathbf{y}^{\text{truth}}, \hat{\mathbf{y}}) = \frac{2\mu_{\mathbf{y}^{\text{truth}}}\mu_{\hat{\mathbf{y}}} + c_1}{\mu^2_{\mathbf{y}^{\text{truth}}} + \mu^2_{\hat{\mathbf{y}}} + c_1}, \tag{15}$$

$$c(\mathbf{y}^{\text{truth}}, \hat{\mathbf{y}}) = \frac{2\sigma_{\mathbf{y}^{\text{truth}}}\sigma_{\hat{\mathbf{y}}} + c_2}{\sigma^2_{\mathbf{y}^{\text{truth}}} + \sigma^2_{\hat{\mathbf{y}}} + c_2}, \tag{16}$$

$$s(\mathbf{y}^{\text{truth}}, \hat{\mathbf{y}}) = \frac{\sigma_{\mathbf{y}^{\text{truth}}\hat{\mathbf{y}}} + c_3}{\sigma_{\mathbf{y}^{\text{truth}}}\sigma_{\hat{\mathbf{y}}} + c_3}. \tag{17}$$

Here, $\mu_{\mathbf{y}^{\text{truth}}}$ and $\mu_{\hat{\mathbf{y}}}$ are the mean values of $\mathbf{y}^{\text{truth}}$ and $\hat{\mathbf{y}}$, $\sigma^2_{\mathbf{y}^{\text{truth}}}$ and $\sigma^2_{\hat{\mathbf{y}}}$ are their variances, and $\sigma_{\mathbf{y}^{\text{truth}}\hat{\mathbf{y}}}$ is the covariance. Constants c_1, c_2, and c_3 are small constants added to stabilize the division with a weak denominator.

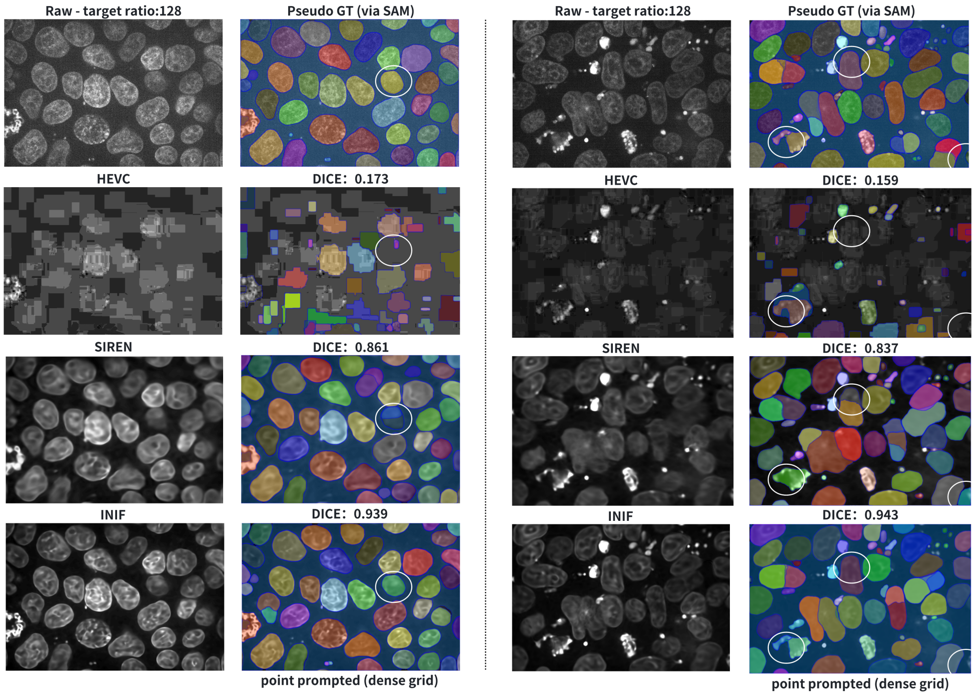

Supplementary Figure 6: **Dense Prompted Result with Segment Anything Model,** To facilitate comparison, we have highlighted the common failure cases in other compression outcomes. Columns 1 and 3 (from left) illustrate the compressed images produced by various compressors when compared to the raw image. Columns 2 and 4 (from left) present the corresponding segmentation results after dense grid prompting. Implicit methods, such as SIREN and INIF, demonstrate superior performance; however, INIF exhibits better handling of corner cases when the edge contrast of nuclei is low.

3 Additional Experiments

3.1 Stress test on larger images

We conducted stress tests on three large 3D TIFF images with the size of 4GB, 11GB, and 23GB, respectively. The 4GB image depicts a whole-mount mouse lung stained with CD3 (T cells) and CD19 (B cells), dehydrated with Ethanol, and cleared with ethyl cinnamate (ECi). The 11GB and 23GB images show the mouse gut injected with CD31 (vessels) and EpCAM (Epithelium) antibodies, then dehydrated with Ethanol and cleared with ECi.

Mouse lung and gut samples were dehydrated with serial ethanol treatment of 20%, 40%, 60%, 80% and 100% twice and cleared with Ethyl cinnamate (ECI, Cat. 112372, Sigma). Afterward, the samples were imaged by LSFM (Ultramicroscope BLAZE, Miltenyi Biotech, Germany). Samples were placed on a steel sample holder and put

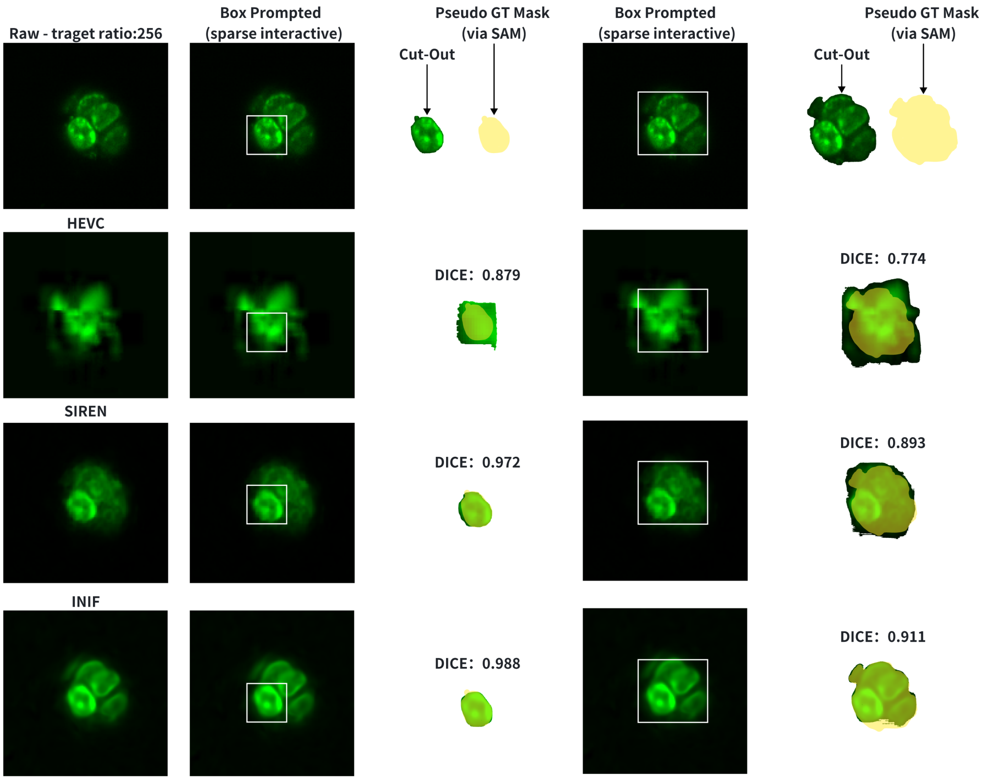

Supplementary Figure 7: **Sparse Prompted Result with Segment Anything Model.** To demonstrate various demand scenarios, we focused on the segmentation of a single cell as well as the separation of foreground and background. The first column (from left) presents the compression result in comparison to the raw image. The second and fourth columns (from left) illustrate the box prompts with varying scales provided to the SAM model. The third and fifth columns (from left) compare the segmentation results. The yellow mask is the pseudo ground truth (GT) mask obtained from the raw image via SAM. The original raw image is then cut out by the segmentation results from either the original image or the compressed images and overlaid with the yellow pseudo GT mask, in order to visualize the consistency between the segmentation results and the pseudo GT over the original image. Our INIF method exhibited the highest consistency across both scenarios, achieving superior DICE scores and visualization fidelity.

in an imaging chamber filled with 100% ECI solution. The light-sheet width has been set to 40% and the numerical aperture was set to 0.05. These settings correspond to a light-sheet thickness of 4 µm. The images were acquired with a 4x objective with an interval of 5 µm.

Due to memory constraints, we partitioned the large image into smaller sections, ensuring each section fits within the memory capacity (we used one 40GB NVIDIA

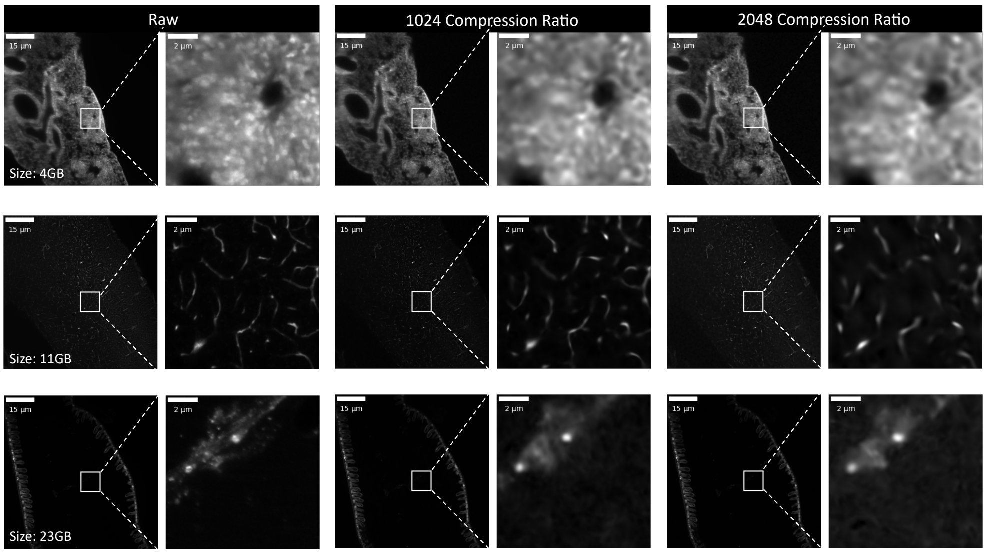

Supplementary Figure 8: **INIF under large tiff images.** INIF demonstrates effective compression performance on large TIFF images. When applying different compression ratios (1024 2048), INIF shows satisfactory results across the selected set of three large TIFF images. A detailed comparison is conducted by zooming in on a specific slice of the image.

A100 GPU). Chunking was performed along the z-axis. Consequently, the INIF algorithm was employed on each chunk utilizing two distinct compression ratios (1024, 2048), respectively. The results are shown in **Supplementary Figure 8**, which verifies the applicability.

3.2 Real world application test with Segment Anything Model

In *prompt-based segmentation* leveraging Segment Anything Model (SAM) [36], the perceptual quality of compressed images plays a pivotal role in enabling users to achieve specific segmentation objectives through prompts. This dual requirement—preserving model perceptual fidelity and human perceptual similarity—represents a unique challenge in real-world scenarios. *Dense grid point prompting* is a well-established technique for performing panoramic segmentation using the SAM model. Each grid intersection point serves as a prompt for SAM, generating a unique output mask. Subsequently, de-duplication is performed among the masks to produce the final mask. Our results under such settings (**Supplementary Figure 6**) illustrate that INIF's compression technique effectively retains SAM's perceptual similarity, leading to accurate and reliable segmentation outcomes, even when compression is applied. This is particularly critical for microscopy images, where even subtle deviations in model perceptual similarity can result in significant segmentation errors, which are also hard to identify by metrics alone.

Furthermore, in *interactive sparse box prompting* scenarios (**Supplementary Figure 7**), which is also the most common usage in SAM. Users can employ three types of prompts (point, box, and mask) to control the masking output according to specific demands. We tested the use of box prompts. INIF shows its capability to avoid severe block artifacts or the oversmoothed blending of adjacent structures during compression, issues commonly observed in competing methods. As a result, INIF ensures segmentation consistency across different scales (i.e, foreground-background, extracting a single cell), allowing users to identify the target prompting area and achieve desired results regardless of the granularity of their segmentation requirement. This is important in applications where maintaining the structural integrity of individual cells is crucial for downstream biological analysis.

3.3 Real examples with sub-optimal performance

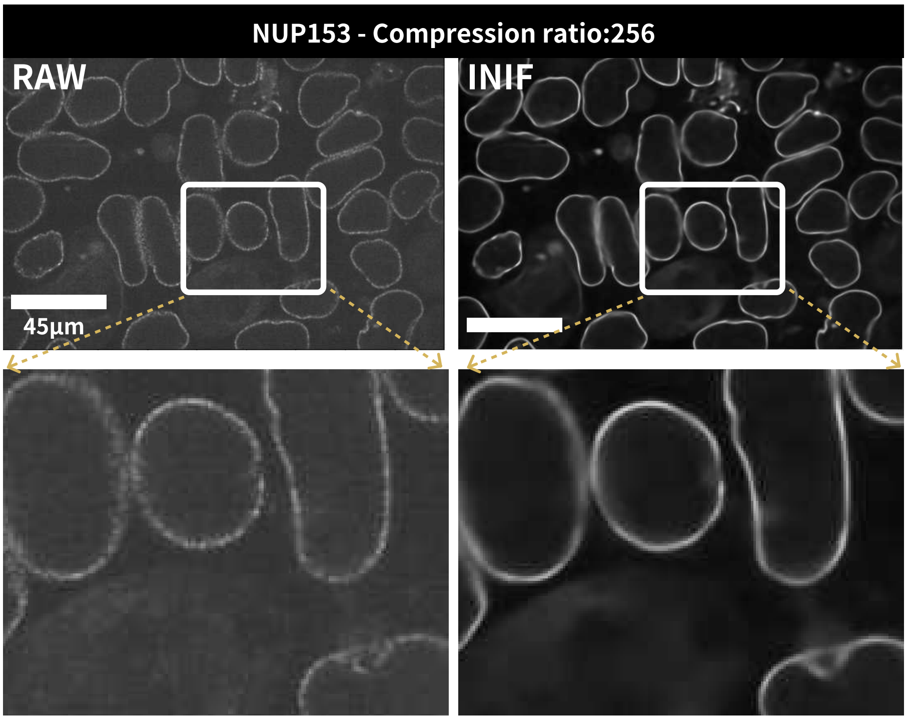

Supplementary Figure 9: **Over-smooth artifacts produced by INR-based compression.** The protein NUP153 is associated with nuclear pores. The structural analysis of raw images indicates that it should exhibit discrete dots arranged around the nuclei. However, INR networks have a tendency to acquire a continuous representation.

Although we consider the INR method as a promising compressor thanks to its flexibility and differentiable nature, it may not always achieve satisfactory results for specific applications. One major drawback of INR-based methods lies in the tendency of neural networks to learn continuous functions, posing a challenge in preventing excessive smoothing even with the INIF workflow. As illustrated in **Supplementary Figure 9**, the compression of microscopy images of nuclear pores sometimes may yield unsatisfactory results. We can observe that the decompressed image deviates from the actual dense "puncta-like" structures. This issue could be alleviated by designing application-specific guidance. For example, in our future work, we could utilize guidance such as segmentation loss to prioritize the preservation of such morphology information.

3.4 Additional Quantitative Evaluation on Compression Benchmarks

To further evaluate INIF towards a broader scene, we followed the benchmark provided in more recent publications (e.g. SCI [37] and TINC [38]). Specifically, the HiPCT dataset [39] used in those two publications provides cellular-level imaging of several organisms at multiple anatomical levels. All the preprocessing remains the same as TINC's setup. From **Supplementary Table 7**, we can see that INIF successfully overcomes the challenge of not partitioning and achieves comparable or better results to other baselines without any hyperparameter tuning. Those inherent advancements support INIF in practical usage, especially when scenarios where scalability matters.

From an energy and environmental perspective, a compressor should aim to maximize the net benefit between the energy consumed during compression and the energy saved post-compression. However, evaluating the 'energy cost' is quite comprehensive, especially for INR-based methods, which are relatively new to the community. Based on our observations, recent works such as Cool-chic [47] and C3 [48] evaluated the Multiplication and Accumulation (MAC) metric during encoding and decoding. This is reasonable since, for CODEC, this metric provides a good approximation of the overall efficiency of the compressor. However, this is not the entire story for differentiable compressors, where iterative training is required. Our comparative experiments have revealed the actual high energy cost associated with excessive data partitioning and auto-regressive encoding approaches (**Supplementary Figure 10**). This high cost not only affects the practical applicability of these methods but also raises concerns about their overall energy efficiency and environmental impact. To delve deeper into the issues faced by INR-based compression methods and why relying solely on MAC is not rational anymore, we decomposed the cost during compression into several components:

1. **Multiplication and Accumulation (MAC) per iteration:**
   - Different compression methods may have vastly different computational complexity per iteration, directly impacting overall performance. The smaller MAC value reflects better basic efficiency of the algorithm for one iteration.
2. **Iteration number per hyperparameter configuration:**

Supplementary Table 7: Compression Performance Comparison on HiPCT Data. We adopted the dataset used in SCI and TINC for a benchmark-level quantitative comparison test. Image shape for all organs is set to 256*256*256, with normalized pixel value to interval [-1, 1]. For all the other detailed setups, please refer to TINC's manuscript.

Method	Brain		Heart		Kidney		Lung		Average	
	PSNR	SSIM	PSNR	SSIM	PSNR	SSIM	PSNR	SSIM	PSNR	SSIM
Compression Ratio 64										
JPEG2000 [40]	46.8100	0.9807	51.0700	0.9948	45.4200	0.9746	43.7900	0.9615	46.7725	0.9779
H.264 [41]	50.0100	0.9902	58.4900	0.9746	48.3800	0.9852	45.9100	0.9749	50.6975	0.9812
HEVC [42]	49.3800	0.9894	54.7400	0.9974	48.0200	0.9847	45.5500	0.9735	49.4225	0.9863
DVC (CVPR19) [43]	44.8700	0.9742	46.4800	0.9896	44.2600	0.9703	42.5100	0.9539	44.5300	0.9720
NeRF (ECCV20) [44]	47.5600	0.9839	50.8000	0.9941	47.4200	0.9816	43.0900	0.9536	47.2175	0.9783
SSF (CVPR20) [45]	48.1700	0.9846	52.2100	0.9963	47.0100	0.9802	43.9800	0.9582	47.8425	0.9798
SIREN (NIPS21) [46]	49.2300	0.9888	54.4400	0.9976	48.2800	0.9849	44.3600	0.9627	49.0775	0.9835
NeRV (NIPS21) [12]	46.1100	0.9803	51.2000	0.9959	46.3500	0.9799	43.3900	0.9578	46.7625	0.9785
SCI (AAAI22) [37]	48.6800	0.9878	54.5200	0.9971	47.5300	0.9828	44.1400	0.9620	48.7175	0.9824
TINC (CVPR23) [38]	49.8700	0.9899	56.6600	0.9981	48.0900	0.9840	44.0100	0.9604	49.6575	0.9831
INIF	50.1100	0.9903	58.7100	0.9988	48.7300	0.9861	45.7900	0.9685	50.8350	0.9859
Compression Ratio 128										
JPEG2000 [40]	43.3300	0.9631	45.7600	0.9841	44.3900	0.9715	41.3200	0.9434	43.7000	0.9655
H.264 [41]	49.6200	0.9893	56.5900	0.9982	48.0900	0.9843	44.8400	0.9680	49.7850	0.9850
HEVC [42]	48.6500	0.9876	53.7100	0.9968	47.4500	0.9828	44.7100	0.9677	48.6300	0.9837
DVC (CVPR19) [43]	43.3000	0.9664	46.6900	0.9907	44.4700	0.9719	42.5700	0.9550	44.2575	0.9710
NeRF (ECCV20) [44]	47.0300	0.9819	48.8300	0.9922	46.7300	0.9792	42.7600	0.9519	46.3375	0.9763
SSF (CVPR20) [45]	48.1000	0.9862	51.6300	0.9957	47.5200	0.9831	44.4400	0.9654	47.9225	0.9826
SIREN (NIPS21) [46]	48.4300	0.9868	53.4900	0.9973	47.8200	0.9833	44.0300	0.9603	48.4425	0.9819
NeRV (NIPS21) [12]	44.7600	0.9737	49.9800	0.9943	45.8600	0.9778	42.8900	0.9539	45.8725	0.9749
SCI (AAAI22) [37]	46.7100	0.9801	52.8000	0.9962	47.1400	0.9816	43.1200	0.9591	47.4425	0.9792
TINC (CVPR23) [38]	47.6900	0.9848	52.1300	0.9901	47.5300	0.9823	43.6100	0.9509	47.7400	0.9770
INIF	49.7900	0.9884	56.8200	0.9983	48.2700	0.9847	44.7800	0.9679	49.9150	0.9848
Compression Ratio 256										
JPEG2000 [40]	41.5100	0.9511	43.2100	0.9819	42.0700	0.9610	40.4400	0.9372	41.8075	0.9578
H.264 [41]	48.4400	0.9870	54.0300	0.9974	47.4100	0.9825	44.0300	0.9623	48.4775	0.9823
HEVC [42]	47.8800	0.9855	52.5600	0.9961	46.9400	0.9809	44.0800	0.9627	47.8650	0.9813
DVC (CVPR19) [43]	44.7800	0.9723	47.6900	0.9902	45.5500	0.9746	42.3400	0.9505	45.0900	0.9719
NeRF (ECCV20) [44]	46.3900	0.9795	47.9600	0.9909	46.6300	0.9791	42.5700	0.9511	46.2200	0.9781
SSF (CVPR20) [45]	47.2000	0.9836	50.6400	0.9948	46.8100	0.9807	45.6300	0.9743	47.5700	0.9834
SIREN (NIPS21) [46]	47.7400	0.9847	51.4800	0.9963	47.5500	0.9823	43.4900	0.9563	47.5650	0.9799
NeRV (NIPS21) [12]	44.1600	0.9719	48.4100	0.9924	45.6500	0.9770	42.5600	0.9523	45.1950	0.9734
SCI (AAAI22) [37]	46.4100	0.9798	49.1000	0.9927	46.4500	0.9794	43.0700	0.9549	46.2575	0.9767
TINC (CVPR23) [38]	47.7800	0.9849	52.0100	0.9967	47.2900	0.9801	43.7100	0.9601	47.6975	0.9805
INIF	48.4900	0.9866	54.7300	0.9976	47.8600	0.9834	44.1500	0.9614	48.8075	0.9823
Compression Ratio 512										
JPEG2000 [40]	37.6200	0.9245	37.7600	0.9459	39.6200	0.9495	36.1500	0.9110	37.7875	0.9327
H.264 [41]	45.8000	0.9825	50.7600	0.9958	45.6600	0.9799	42.7100	0.9573	46.2325	0.9789
HEVC [42]	47.1500	0.9830	51.1700	0.9949	46.4900	0.9792	43.5800	0.9585	47.0975	0.9789
DVC (CVPR19) [43]	42.3600	0.9640	44.0800	0.9838	42.8700	0.9700	41.6700	0.9493	42.7450	0.9668
NeRF (ECCV20) [44]	45.6500	0.9770	45.2600	0.9865	46.6200	0.9791	42.4400	0.9506	44.9925	0.9733
SSF (CVPR20) [45]	42.5300	0.9517	46.1600	0.9870	45.4500	0.9751	42.5100	0.9517	44.1625	0.9664
SIREN (NIPS21) [46]	46.8500	0.9818	48.8100	0.9946	47.1100	0.9807	43.1700	0.9546	46.4850	0.9779
NeRV (NIPS21) [12]	44.0700	0.9712	43.6600	0.9849	45.3600	0.9759	42.0000	0.9495	43.7725	0.9704
SCI (AAAI22) [37]	46.2400	0.9807	48.6700	0.9916	45.8500	0.9781	42.5400	0.9511	45.8250	0.9754
TINC (CVPR23) [38]	47.0100	0.9821	51.2100	0.9898	47.0700	0.9807	43.0600	0.9558	47.0875	0.9771
INIF	48.4900	0.9831	51.9400	0.9962	47.4000	0.9818	43.7700	0.9587	47.9000	0.9800

- For training-based methods (i.e. INR-based method), more iterations lead to longer total computation time, especially for large-scale data; the cost for one iteration would be even higher (because of using a larger MLP). For the non-trainable-based method, the cost equals the per-iteration MAC. This is also the main reason why CODEC can be much cheaper. Because they are undifferentiated, and there is no need for training.

3. **Hyperparameter search cost per Partition:**
   - Except for INIF, other methods may require multiple trials to search for reasonable hyperparameters for each partition of the full data, affecting overall

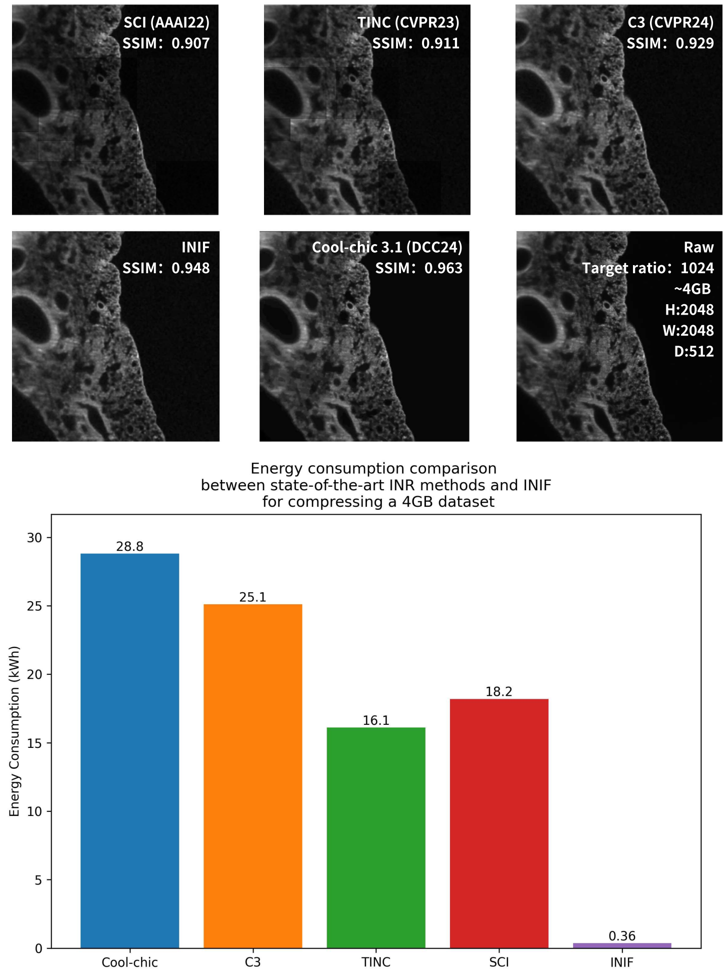

Supplementary Figure 10: Energy consumption and compression quality comparison to the state-of-the-art INR-based compression methods on large microscopy data. All methods are tested on a single A100 40GB GPU with around 90% usage rate during training. INIF show 70-fold less energy usage compared to Cool-chic 3.1 but remains a comparable outcome.

efficiency. For instance, we have searched for the Bitrate, Constant Rate Factor (CRF), and Adaptive Quantization (AQ) mode when using CODEC, learning rate, Group of Picture (GOP) strategies, for Cool-chic and C3.

4. **Partition number per sample:**

- Most of the lossy compressors, including CODEC and the majority of neural compressors, are all designed for 2D-3D natural images. For High-dimensional

Supplementary Table 8: **Experimental setup**: Details for benchmark-level test using HiPSC multichannel 3D samples are listed.

Dataset	Image Size	Sample #	Cell Lines #	Batch Size	Device
HiPSC [49]	$4, 65, 650, 920$	25	25	100000	Single RTX4090

biomedical data, using those methods may require partitioning strategies, constituting a significant computational cost factor. For instance, in our 5D data test, we have to split the original data into 270 partitions when using a 3D compressor such as HEVC.

5. **Decode cost:**

- Decoding speed is also important in certain application scenarios, especially for frequently accessed data. However, the major cost comes from the encoding part. Notice that the decoding strategy depends on the usage of data; the flexibility and convenience of decoding should be the key factor when evaluating the general cost for decoding in practice.

For those reasons, we propose a comprehensive cost calculation formula:

$$\text{Encode cost} = \text{MAC} \times \text{Iter} \times \text{Hyper} \times \text{Part} \tag{18}$$

$$\text{Overall cost} = \text{Encode cost} + \text{Decode cost} \tag{19}$$

With this formula, we can reveal the true bottlenecks which limit the INR-based compressor's efficiency, and why we consider using a learned optimizer could become an elegant solution for all these bottlenecks. See detailed discussions below.

3.5 Ablation Experiments for the Effect of Learned Optimizer & CODEC Prior Guidance on Training Costs

The biggest bottleneck for all INR-based methods is probably the cost when training iteratively; this is more severe when attempting to overfit the sample, since it usually requires more iterative steps. The learned optimizer backbone we used is the Versatile Learned Optimizer (VeLO) [50]. From VeLO's original technical report, Figure 1, the authors stated: "On 83 canonical tasks in the VeLOdrome benchmark, we train all tasks faster than learning rate-tuned Adam. On about half of the tasks, we are more than 4x faster than learning rate-tuned Adam. On more than 14% of the tasks, we are more than 16x times faster." This dramatic reduction in iteration number motivates us to use VeLO for INR-based compression tasks further. We have also experimented to evaluate this reduction. Under the experimental setup in the **Supplementary Table 8**, we have observed a much faster convergent speed when using the learned optimizer. Additionally, we also introduced the concept of CODEC's prior guidance as an optional novel design. The main contribution of CODEC's prior guidance in our INIF framework is to develop a mutual improvement pipeline by incorporating INIF as an adapter and equipping CODEC with the ability to adjust its learning

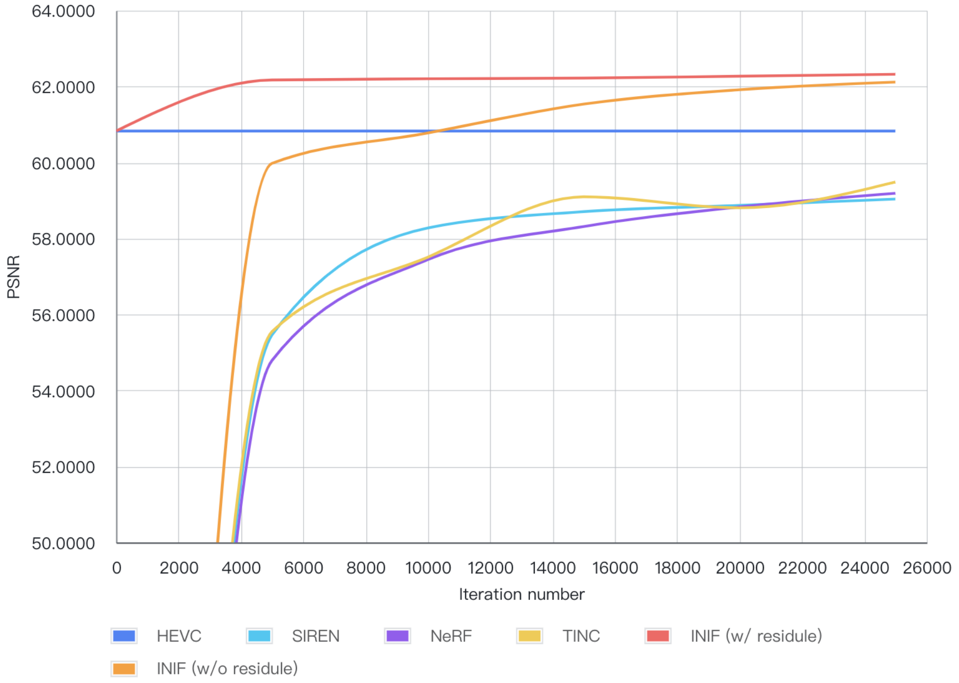

Supplementary Figure 11: **Convergent speed comparison between different methods under compression ratio 256.** INIF uses a learned optimizer to accelerate the convergent speed; this can be further integrated with CODEC prior guidance to serve as a post-compression adapter with high efficiency.

objective freely; meanwhile, CODEC can also boost the compression speed for INIF. (**Supplementary Figure** 11). We believe this behaviour sheds light on a plausible solution for narrowing the gap between the iteration cost of CODEC and INR-based compressors.

3.6 Ablation Experiments for the Effect of Learned Optimizer on Hyperparameter Tuning costs

Another cost which can be significantly reduced by a learned optimizer is the cost of searching hyperparameters. This cost is important in compressing tasks since we unavoidably need a set of hyperparameters for each sample, no matter whether using CODEC or INR to get a close-to-optimal outcome. However, this cost is often ignored. For CODEC, a range of hyperparameters controls the compression ratio and affects the final quality. For example, we have searched for the Bitrate, Constant Rate Factor (CRF), and Adaptive Quantization (AQ) mode when using CODEC. For INR-based methods, the basic hyperparameter for all the baselines is the learning rate (LR), and

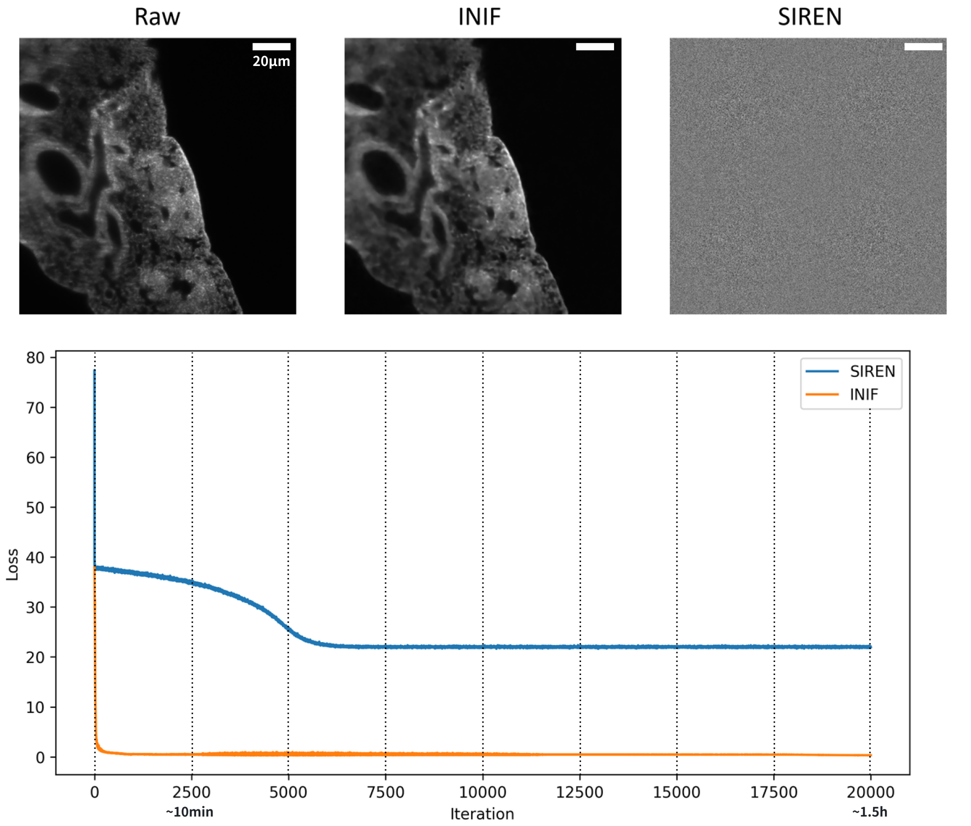

Supplementary Figure 12: **Comparison of INIF and SIREN performance on stress test.** Raw data (top left) and compressed results (top middle, right). The SIREN decompressed image demonstrates a case where inappropriate hyperparameters led to nonsensical results, highlighting the importance of proper parameter tuning in methods optimized by hand-crafted optimizers. The Loss curves for INIF and SIREN during training (bottom) further reveal the hidden cost parameter tuning. Note that even with nonsensical results, both methods show decreasing loss.

additional hyperparameters for each method specifically, e.g., GOP mode for Cool-chic, partition number for TINC, etc. We have demonstrated that improper LR would severely affect the final compression quality. This would be a bigger issue when compressing large data. For example, using the 4GB data in **Supplementary Figure** 10. The result by SIREN with a default learning rate of 0.001 with cosine annealing scheduling is undesired (**Supplementary Figure** 12). Nevertheless, without visualization or comparison to other baselines, we cannot have a clear judgment of the quality from the plotted loss curve alone. In this case, the cost of searching a set of reasonable hyperparameters would be fairly high because this requires multiple search

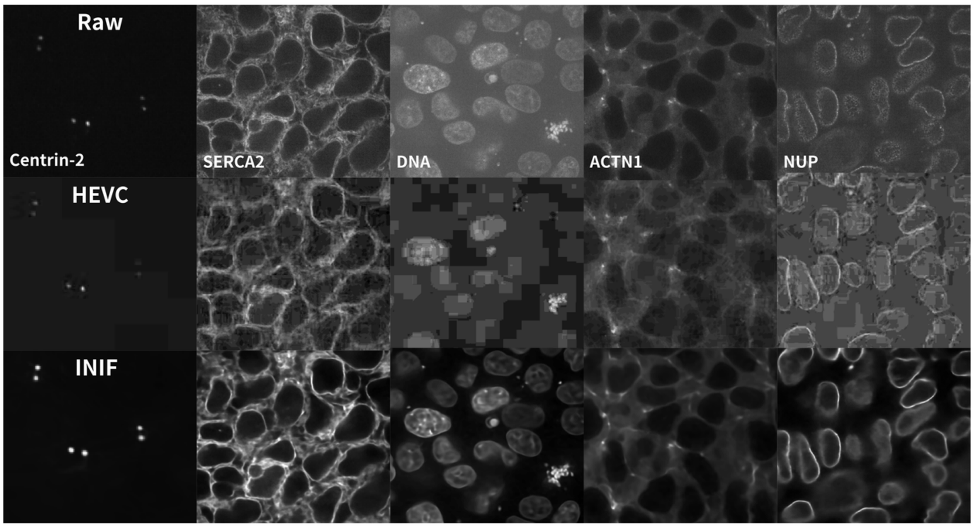

Supplementary Figure 13: Comparison of raw microscopy images with HEVC and INIF compression results. Confocal microscopy images of five different organelles are shown on the top row: centrioles (marked via Centrin-2), ER (SERCA) (marked via SERVAA2), DNA, actin bundles(marked via ACTN1), nuclear pores (marked via Nup153).

trials. INIF, in contrast, uses a learned optimizer to search for the optimal optimization direction and step size automatically. Which does not need to set a hard hyperparameter, such as the learning rate, before training. All the results shown are based on a single trial; this could be considered a very convenient feature for further propagating INR-based compressors as a usable tool to the community.

3.7 Ablation Experiments for the Effect of Learned Optimizer & Application Appropriate Guidance on Partitioning costs

The final reason for using a learned optimizer is to preserve the unique attributes of INR to the maximum extent. While we acknowledge that the partition strategy adopted by most of the state-of-the-art INR compressors would be one of the solutions, the inconvenience during encoding and decoding in practical usage is also evident. Considering the data dimension and pixel depth of biomedical data often exceeds the regular 2D, 3D, and 8-bit fashion. The issue, along with partitioning, becomes more severe. Under this setup, we discussed 3 compression scenarios. In the first scenario, when the data structure is suitable for CODEC and the compression quality of CODEC is acceptable, we recommend incorporating CODEC, as they are sufficient for most applications. Nevertheless, we enhance CODEC's boundary by introducing residual compression with an INIF network to further improve compression quality. Our application-appropriate loss guidance serves as a post-CODEC adapter for specific user requirements that cannot be easily addressed by an undifferentiable CODEC.

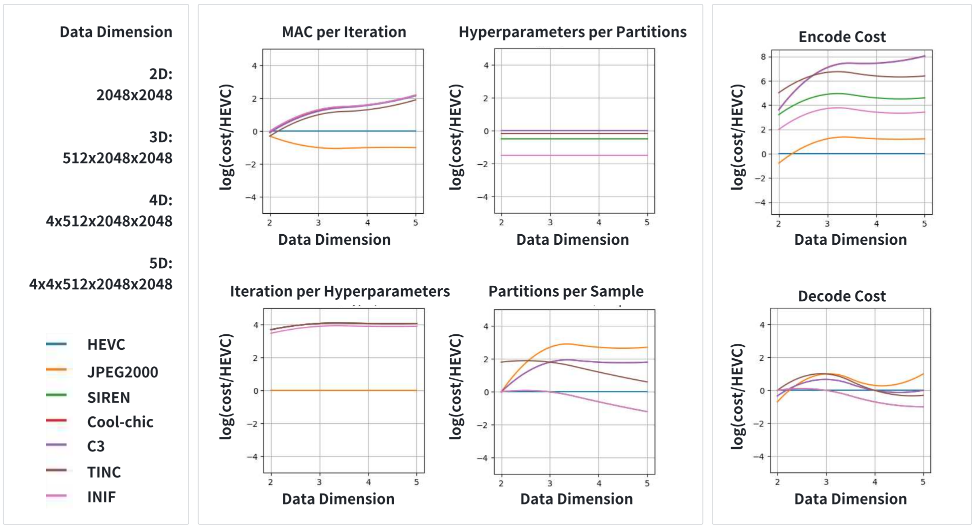

Supplementary Figure 14: Compression performance comparison of various methods across different data dimensions. The graphs illustrate the relative costs of different methods over HEVC. INIF demonstrates consistent performance across dimensions, particularly excelling in high-dimensional scenarios.

Moreover, with biomedical images, the rest of the scenarios are very common, and we believe INR-based methods have better potential to handle the challenges:

1. **Scenario 1: Low-dimensional data with unacceptable CODEC quality** In situations where CODEC quality is unacceptable, which occurs frequently in microscopy imaging (**Supplementary Figure 13**), considering the speed advantage of CODEC becomes pointless; alternative compression strategies become necessary. INIF presents a viable option in these scenarios, offering a balance between compression quality and speed.
2. **Scenario 2: High-dimensional data** For data dimensions greater than 3, INIF, unlike CODEC, neural CODEC, and INR-based methods, heavily relies on partitions; it is designed to handle high-dimensional data natively. This capability is crucial in biomedical imaging, where high-dimensional, high-resolution, and high pixel-depth data are common.

To give a more direct comparison, we provided the cost of different methods under different data dimensions in **Supplementary Figure 14**. INIF narrows the gap in terms of cost with HEVC by all the aforementioned advantages. We further summarized those observations in **Supplementary Table 9**.

Experimentally, we also see INIF hold a great balance between performance and network size without partitioning. We have provided the quantitative results of all the experiments in our main text in **Supplementary Table 6**. From the experiments,

Supplementary Table 9: **Comparison of Compression Methods Across Different Data Dimensions**: A summary of (1) the number of multiplication and accumulation (MAC) per iteration, (2) the number of iterations per hyperparameter, (3) the number of hyperparameters per partition, (4) the number of partition per sample, and (4) decoding cost, of INIF and baseline methods on 2D - 5D data indicate the efficiency of INIF.

Data Dim	Metric	HEVC [42]	JPEG2000 [40]	SIREN (NIPS21) [46]	Cool-chic (DCC24) [47]	C3 (CVPR24) [48]	TINC (CVPR23) [38]	INIF (w/o CODEC prior)
2D	MAC per Iteration	Low	Very Low	High	High	High	High	High
	Iterations per Hyperparameter	1	1	Mid	Mid	Mid	Mid	Mid
	Hyperparameters per Partition	Very Low	Very Low	Low	Low	Low	Low	Very Low
	Partitions per Sample	1	1	1	1	1	Low	1
	Decode	Mid	Mid	Mid	Low	Low	Mid	Mid
3D	MAC per Iteration	Low	Very Low	High	High	High	High	High
	Iterations per Hyperparameter	1	1	High	High	High	High	Mid
	Hyperparameters per Partition	Low	Mid	Low	Mid	Mid	Mid	Very Low
	Partitions per Sample	1	High	Very Low	Mid	Mid	Mid	Very Low
	Decode	Mid	High	Mid	Low	Low	Mid	Mid
4D	MAC per Iteration	Low	Very Low	High	High	High	High	High
	Iterations per Hyperparameter	1	1	High	High	High	High	Mid
	Hyperparameters per Partition	High	Very High	Mid	High	High	Mid	Very Low
	Partitions per Sample	High	Very High	Very Low	High	High	High	Very Low
	Decode	High	Very High	Mid	High	High	High	Mid
5D	MAC per Iteration	Low	Very Low	High	High	High	High	High
	Iterations per Hyperparameter	1	1	High	High	High	High	Mid
	Hyperparameters per Partition	Very High	Very High	High	Very High	Very High	Very High	Very Low
	Partitions per Sample	Very High	Very High	Low	Very High	Very High	Very High	Low
	Decode	Very High	Very High	Mid	Very High	Very High	Very High	Mid

we can see that INIF successfully overcomes the challenge of not partitioning. However, we did not include all these quantitative results in our main text. The main reason is that using simple metrics such as PSNR or SSIM often contains bias and can be misleading when dealing with biomedical data. For example, in the application-appropriate guidance test, we found that relying on PSNR or SSIM alone does not accurately reflect the actual performance in downstream tasks (such as the Dice score). Moreover, it is quite common to see biomedical images taken under specific microscope setups, e.g., varying laser power. When evaluating such data, naive metrics are not robust. This is also an important topic often discussed by the biomedical imaging community. Recent publications have also pointed out that such a phenomenon is widespread across different tasks, not only in compression [51, 52].

Theoretically, we also aim to explore the potential of learned optimizers and application-specific guidance as a viable solution for mitigating underfitting issues without the need for partitioning. From an optimization perspective, the objective for any INR network can be reformulated as learning the function $f(\theta)$ parameterized by a neural network and mapping spatiotemporal coordinate space (x, y, z) (or any other implicit representation of data) to pixel intensity space (R, G, B) (or any other explicit data value). This mapping process can be formulated as:

$$f(x, y, z, \theta) = (R, G, B) \tag{20}$$

From an information theory perspective, INR networks essentially perform entropy decoding. The input coordinates (x, y, z) have relatively low entropy due to their inherent spatiotemporal structure and regularity. The INR network then decodes this low-entropy input into high-entropy output data (R, G, B), which typically contains more complex and varied information. This process can be seen as a form of data expansion or decompression, where the network learns to reconstruct complex, high-entropy data from simple, low-entropy inputs.

When utilizing INR for compression purposes, we introduce an additional constraint to limit the size of θ. This constraint ensures that the network parameters are compressed. The optimization problem can then be formulated as:

$$\begin{aligned} \min_{\theta} \quad & \mathcal{L}(f(x,y,z,\theta),(R,G,B)) \\ \text{s.t.} \quad & \|\theta\| \leq C \end{aligned} \tag{21}$$

where $\mathcal{L}$ is a loss function measuring the difference between the predicted and true values, and C is a constant that limits the size or complexity of the network parameters θ. Both $\mathcal{L}$ and C can be implemented in various ways, such as $\mathcal{L}$ can be naively Mean Square Error (MSE) to target pixel level reconstruction, or it can also be the application-appropriate losses we proposed in the manuscript. C can be used to limit the number of parameters, apply weight quantization, or use regularization techniques. The introduction of parameter constraints also transforms our optimization problem into a search for Pareto Optimality. This further increases the complexity of the optimization process. The problem can be reframed as a multi-objective optimization:

$$\min_{\theta} \quad (\mathcal{L}(f(x,y,z,\theta),(R,G,B)), \|\theta\|) \tag{22}$$

where we seek to minimize both the reconstruction error and the parameter size simultaneously. This formulation explicitly highlights the trade-off between these two objectives, and the set of optimal solutions forms a Pareto front.

Traditional hand-crafted optimizers, which strictly adhere to gradient descent strategies, often struggle with this type of constrained optimization scenario. Once parameters reach a local optimum, it becomes challenging to make significant updates. However, dealing with constrained optimization typically requires continuous exchange between optima or even reparameterization. In this context, learned optimizers offer an alternative. They can, to some extent, ignore the limitations of gradients and make larger updates. A learned optimizer can be formulated as:

$$\theta_{t+1} = \theta_t + g(\nabla \mathcal{L}, \theta_t, \phi) \tag{23}$$

where g is a learned update rule parameterized by ϕ, unlike traditional optimizers that use fixed update rules, learned optimizers can adapt their behaviour based on the optimization landscape and the history of updates. This flexibility allows them to have the theoretical potential to escape local optima and explore the Pareto front more effectively.

References

[1] Chen, Y., Liu, S. & Wang, X. Learning continuous image representation with local implicit image function 8628–8638 (2021).

[2] Zhao, J. *et al.* Moec: Mixture of experts implicit neural compression. *arXiv preprint arXiv:2312.01361* (2023).

[3] Sitzmann, V., Martel, J., Bergman, A., Lindell, D. & Wetzstein, G. Implicit neural representations with periodic activation functions. *Advances in neural information processing systems* **33**, 7462–7473 (2020).

[4] Mildenhall, B. *et al.* Nerf: Representing scenes as neural radiance fields for view synthesis. *Communications of the ACM* **65**, 99–106 (2021).

[5] Thies, J., Zollhöfer, M. & Nießner, M. Deferred neural rendering: Image synthesis using neural textures. *Acm Transactions on Graphics (TOG)* **38**, 1–12 (2019).

[6] Chen, H. *et al.* Nerv: Neural representations for videos **34**, 21557–21568 (2021). URL https://proceedings.neurips.cc/paper_files/paper/2021/file/b44182379bf9fae976e6ae5996e13cd8-Paper.pdf.

[7] Xian, W., Huang, J.-B., Kopf, J. & Kim, C. Space-time neural irradiance fields for free-viewpoint video 9421–9431 (2021).

[8] Su, K., Chen, M. & Shlizerman, E. Inras: Implicit neural representation for audio scenes. *Advances in Neural Information Processing Systems* **35**, 8144–8158 (2022).

[9] Lanzendörfer, L. A. & Wattenhofer, R. Siamese siren: Audio compression with implicit neural representations. *arXiv preprint arXiv:2306.12957* (2023).

[10] Strümpler, Y., Postels, J., Yang, R., Gool, L. V. & Tombari, F. Implicit neural representations for image compression 74–91 (2022).

[11] Popescu, M.-C., Balas, V. E., Perescu-Popescu, L. & Mastorakis, N. Multilayer perceptron and neural networks. *WSEAS Transactions on Circuits and Systems* **8**, 579–588 (2009).

[12] Chen, H. *et al.* Nerv: Neural representations for videos. *Advances in Neural Information Processing Systems* **34**, 21557–21568 (2021).

[13] Fathony, R., Sahu, A. K., Willmott, D. & Kolter, J. Z. Multiplicative filter networks (2020).

[14] Lu, Y., Jiang, K., Levine, J. A. & Berger, M. Compressive neural representations of volumetric scalar fields **40**, 135–146 (2021).

[15] Klocek, S. *et al.* Hypernetwork functional image representation 496–510 (2019).

[16] Mehta, I. *et al.* Modulated periodic activations for generalizable local functional representations 14214–14223 (2021).

[17] Benbarka, N., Höfer, T., Zell, A. *et al.* Seeing implicit neural representations as fourier series 2041–2050 (2022).

[18] Müller, T., Evans, A., Schied, C. & Keller, A. Instant neural graphics primitives with a multiresolution hash encoding. *ACM transactions on graphics (TOG)* **41**, 1–15 (2022).

[19] Bengio, S., Bengio, Y., Cloutier, J. & Gecsei, J. On the optimization of a synaptic learning rule (2002).

[20] Runarsson, T. P. & Jonsson, M. T. Evolution and design of distributed learning rules 59–63 (2000).

[21] Andrychowicz, M. *et al.* Learning to learn by gradient descent by gradient descent. *Advances in neural information processing systems* **29** (2016).

[22] Wichrowska, O. *et al.* Learned optimizers that scale and generalize 3751–3760 (2017).

[23] Metz, L., Maheswaranathan, N., Freeman, C. D., Poole, B. & Sohl-Dickstein, J. Tasks, stability, architecture, and compute: Training more effective learned optimizers, and using them to train themselves. *arXiv preprint arXiv:2009.11243* (2020).

[24] Metz, L. *et al.* Using a thousand optimization tasks to learn hyperparameter search strategies. *arXiv preprint arXiv:2002.11887* (2020).

[25] Metz, L. *et al.* Velo: Training versatile learned optimizers by scaling up. *arXiv preprint arXiv:2211.09760* (2022).

[26] Wiegand, T., Sullivan, G. J., Bjontegaard, G. & Luthra, A. Overview of the H.264/AVC video coding standard. *IEEE Transactions on circuits and systems for video technology* **13**, 560–576 (2003).

[27] Sullivan, G. J., Ohm, J.-R., Han, W.-J. & Wiegand, T. Overview of the high efficiency video coding (HEVC) standard. *IEEE Transactions on circuits and systems for video technology* **22**, 1649–1668 (2012).

[28] Gao, Y., Zhu, C., Li, S. & Yang, T. Temporally dependent rate-distortion optimization for low-delay hierarchical video coding. *IEEE Transactions on Image Processing* **26**, 4457–4470 (2017).

[29] Cassa, M. B., Naccari, M. & Pereira, F. Fast rate distortion optimization for the emerging hevc standard 493–496 (2012).

[30] Li, B., Li, H., Li, L. & Zhang, J. λ domain rate control algorithm for high efficiency video coding. *IEEE Transactions on Image Processing* **23**, 3841–3854 (2014).

[31] Patro, S. & Sahu, K. K. Normalization: A preprocessing stage. *arXiv preprint arXiv:1503.06462* (2015).

[32] Johnson, J., Alahi, A. & Fei-Fei, L. Perceptual losses for real-time style transfer and super-resolution 694–711 (2016).

[33] Krizhevsky, A., Sutskever, I. & Hinton, G. E. Imagenet classification with deep convolutional neural networks. *Advances in neural information processing systems* **25** (2012).

[34] Chen, J. *et al.* The allen cell and structure segmenter: a new open source toolkit for segmenting 3d intracellular structures in fluorescence microscopy images. *BioRxiv* 491035 (2018).

[35] Wang, Z., Bovik, A. C., Sheikh, H. R. & Simoncelli, E. P. Image quality assessment: from error visibility to structural similarity. *IEEE transactions on image processing* **13**, 600–612 (2004).

[36] Kirillov, A. *et al.* *Segment anything*, 4015–4026 (2023).

[37] Yang, R. *et al.* Sci: A spectrum concentrated implicit neural compression for biomedical data. *ArXiv* **abs/2209.15180** (2022). URL https://api.semanticscholar.org/CorpusID:252668529.

[38] Yang, R., Xiao, T., Cheng, Y., Suo, J. & Dai, Q. Tinc: Tree-structured implicit neural compression. *2023 IEEE/CVF Conference on Computer Vision and Pattern Recognition (CVPR)* 18517–18526 (2022). URL https://api.semanticscholar.org/CorpusID:253510838.

[39] Walsh, C. *et al.* Imaging intact human organs with local resolution of cellular structures using hierarchical phase-contrast tomography. *Nature methods* **18**, 1532–1541 (2021).

[40] Christopoulos, C., Skodras, A. & Ebrahimi, T. The jpeg2000 still image coding system: an overview. *IEEE transactions on consumer electronics* **46**, 1103–1127 (2000).

[41] Wiegand, T., Sullivan, G. J., Bjontegaard, G. & Luthra, A. Overview of the h. 264/avc video coding standard. *IEEE Transactions on circuits and systems for video technology* **13**, 560–576 (2003).

[42] Sze, V., Budagavi, M. & Sullivan, G. J. High efficiency video coding (hevc) **39**, 40 (2014).

[43] Lu, G. *et al.* *Dvc: An end-to-end deep video compression framework*, 11006–11015 (2019).

[44] Mildenhall, B. *et al.* *Nerf: Representing scenes as neural radiance fields for view synthesis*, 405–421 (Springer, 2020).

[45] Agustsson, E. *et al. Scale-space flow for end-to-end optimized video compression*, 8503–8512 (2020).

[46] Sitzmann, V., Martel, J. N., Bergman, A. W., Lindell, D. B. & Wetzstein, G. Implicit neural representations with periodic activation functions. *Advances in Neural Information Processing Systems* **33**, 7462–7473 (2020).

[47] Blard, T. *et al.* Overfitted image coding at reduced complexity. *arXiv preprint arXiv:2403.11651* (2024).

[48] Kim, H., Bauer, M., Theis, L., Schwarz, J. R. & Dupont, E. C3: High-performance and low-complexity neural compression from a single image or video 9347–9358 (2024).

[49] Viana, M. P. *et al.* Integrated intracellular organization and its variations in human ips cells. *Nature* **613**, 345–354 (2023).

[50] Metz, L. *et al.* Velo: Training versatile learned optimizers by scaling up. *arXiv preprint arXiv:2211.09760* (2022).

[51] Reinke, A. *et al.* Understanding metric-related pitfalls in image analysis validation. *Nature methods* **21**, 182–194 (2024).

[52] Chen, J., Viana, M. P. & Rafelski, S. M. When seeing is not believing: application-appropriate validation matters for quantitative bioimage analysis. *Nature Methods* **20**, 968–970 (2023).
