# Supplementary material for: Implicit neural image field for biological microscopy image compression
Source: Nat Comput Sci. 2025 Oct 10;5(11):1041–50. doi: 10.1038/s43588-025-00889-4 (PMC12638249; doi:10.1038/s43588-025-00889-4)
Supplement: Supplementary file 2 — Reporting Summary [file 43588_2025_889_MOESM2_ESM.pdf]

nature portfolio

Corresponding author(s): Jianxu Chen
Last updated by author(s): Jun 11, 2024

Reporting Summary

Nature Portfolio wishes to improve the reproducibility of the work that we publish. This form provides structure for consistency and transparency in reporting. For further information on Nature Portfolio policies, see our Editorial Policies and the Editorial Policy Checklist.

Statistics

For all statistical analyses, confirm that the following items are present in the figure legend, table legend, main text, or Methods section.

n/a	Confirmed	
☐	☒	The exact sample size (*n*) for each experimental group/condition, given as a discrete number and unit of measurement
☐	☒	A statement on whether measurements were taken from distinct samples or whether the same sample was measured repeatedly
☒	☐	The statistical test(s) used AND whether they are one- or two-sided<br>*Only common tests should be described solely by name; describe more complex techniques in the Methods section.*
☒	☐	A description of all covariates tested
☒	☐	A description of any assumptions or corrections, such as tests of normality and adjustment for multiple comparisons
☐	☒	A full description of the statistical parameters including central tendency (e.g. means) or other basic estimates (e.g. regression coefficient) AND variation (e.g. standard deviation) or associated estimates of uncertainty (e.g. confidence intervals)
☒	☐	For null hypothesis testing, the test statistic (e.g. *F*, *t*, *r*) with confidence intervals, effect sizes, degrees of freedom and *P* value noted<br>*Give P values as exact values whenever suitable.*
☒	☐	For Bayesian analysis, information on the choice of priors and Markov chain Monte Carlo settings
☒	☐	For hierarchical and complex designs, identification of the appropriate level for tests and full reporting of outcomes
☒	☐	Estimates of effect sizes (e.g. Cohen's *d*, Pearson's *r*), indicating how they were calculated

Our web collection on statistics for biologists contains articles on many of the points above.

Software and code

Policy information about availability of computer code

Data collection	All data used in the main text are from public data repositories, e.g., from previous publications.
Data analysis	all data being analyzed in the manuscript were performed using the provided code (shared via Github, https://github.com/PKU-HMI/INIF)

For manuscripts utilizing custom algorithms or software that are central to the research but not yet described in published literature, software must be made available to editors and reviewers. We strongly encourage code deposition in a community repository (e.g. GitHub). See the Nature Portfolio guidelines for submitting code & software for further information.

Data

Policy information about availability of data

All manuscripts must include a data availability statement. This statement should provide the following information, where applicable:

- Accession codes, unique identifiers, or web links for publicly available datasets
- A description of any restrictions on data availability
- For clinical datasets or third party data, please ensure that the statement adheres to our policy

All data used in the main text are from public data repositories, e.g., from previous publications. We have included the data availability section in the manuscript to provide all details.

Human research participants

Policy information about studies involving human research participants and Sex and Gender in Research.

Reporting on sex and gender	n/a
Population characteristics	n/a
Recruitment	n/a
Ethics oversight	n/a

Note that full information on the approval of the study protocol must also be provided in the manuscript.

Field-specific reporting

Please select the one below that is the best fit for your research. If you are not sure, read the appropriate sections before making your selection.

☒ Life sciences ☐ Behavioural & social sciences ☐ Ecological, evolutionary & environmental sciences

For a reference copy of the document with all sections, see nature.com/documents/nr-reporting-summary-flat.pdf

Life sciences study design

All studies must disclose on these points even when the disclosure is negative.

Sample size	All training were done per sample due to the specific algorithm developed in the paper, i.e., the same displayed in the manuscript are the entire sample collection we used, as our compression method will train one model per sample.
Data exclusions	no
Replication	we provide full details in the repository to reproduce our results shown in the paper.
Randomization	Samples were not randomized, as single samples were used to generate algorithm training data. The selection of samples from the public data repository was random.
Blinding	Investigators were not blinded to sample specifications, as the experiments do not include sample group comparisons.

Reporting for specific materials, systems and methods

We require information from authors about some types of materials, experimental systems and methods used in many studies. Here, indicate whether each material, system or method listed is relevant to your study. If you are not sure if a list item applies to your research, read the appropriate section before selecting a response.

Materials & experimental systems

n/a	Involved in the study	
☐	☒	Antibodies
☒	☐	Eukaryotic cell lines
☒	☐	Palaeontology and archaeology
☐	☒	Animals and other organisms
☒	☐	Clinical data
☒	☐	Dual use research of concern

Methods

n/a	Involved in the study	
☒	☐	ChIP-seq
☒	☐	Flow cytometry
☒	☐	MRI-based neuroimaging

Antibodies

Antibodies used

This information is only related to the stress test in the supplementary information (Appendix C.1)
Light sheet fluorescence microscopy:
CD31, MEC13.3 clone, Biolegend, Cat. No:102528, 1:100
EpCAM (CD326), G8.8 clone, Biolegend, Cat. No:118212, 1:100

	CD3, 17A2 clone, Biolegend, Cat. No:100209, 1:100<br>CD19, REAfinity, Miltenyi Biotec, Cat. No:130-131-141, 1:50
Validation	This information is only related to the stress test in the supplementary information (Appendix C.1)<br>All antibodies were bought from commercial suppliers as stated in the reporting summary. Widely used antibody clones and companies were selected, single- and multi-color stainings were made to verify filter settings during imaging. Validations supplied by the manufacturers are also available in the respective websites reached by the catalog numbers.

Animals and other research organisms

Policy information about studies involving animals; ARRIVE guidelines recommended for reporting animal research, and Sex and Gender in Research

Laboratory animals	C57/BL6/J 8-12 weeks old wildtype mice for LSFM imaging experiments. Mice were housed in individually ventilated cages with a 12-hour dark/night cycle. Housing rooms have 21-23°C temperature and 40-60% humidity.
Wild animals	The study did not use wild animals.
Reporting on sex	Only female animals were used for LSFM imaging studies.
Field-collected samples	The study did not use field-collected samples.
Ethics oversight	Ethical approvals were obtained from the local authority, Landesamt für Natur, Umwelt und Verbraucherschutz Nordrhein-Westfalen, under permission number G1719/19 and conducted in accordance with the ARRIVE guidelines.

Note that full information on the approval of the study protocol must also be provided in the manuscript.
